# Transforming growth factor beta 1 mediates the low-frequency vertical vibration enhanced production of tenomodulin and type I collagen in rat Achilles tendon

**DOI:** 10.1371/journal.pone.0205258

**Published:** 2018-10-11

**Authors:** Chia-Hsin Chen, Yi-Hsiung Lin, Chung-Hwan Chen, Yan-Hsiung Wang, Ming-Long Yeh, Tsung-Lin Cheng, Chau-Zen Wang

**Affiliations:** 1 Department of Physical Medicine and Rehabilitation, Kaohsiung Medical University Hospital, Kaohsiung, Taiwan; 2 Department of Physical Medicine and Rehabilitation, Faculty of Medicine, College of Medicine, Kaohsiung Medical University, Kaohsiung, Taiwan; 3 Orthopaedic Research Center, Kaohsiung Medical University, Kaohsiung, Taiwan; 4 Department of Physiology, College of Medicine, Kaohsiung Medical University, Kaohsiung, Taiwan; 5 Graduate Institute of Medicine, College of Medicine, Kaohsiung Medical University, Kaohsiung, Taiwan; 6 Department of Orthopaedics, College of Medicine, Kaohsiung Medical University, Kaohsiung, Taiwan; 7 Department of Orthopedics, Kaohsiung Municipal Ta-Tung Hospital, Kaohsiung Medical University, Kaohsiung, Taiwan; 8 Division of Adult Reconstruction Surgery, Department of Orthopedics, Kaohsiung Medical University Hospital, Kaohsiung Medical University, Kaohsiung, Taiwan; 9 School of Dentistry, Kaohsiung Medical University, Kaohsiung, Taiwan; 10 Department of Medical Research, Kaohsiung Medical University Hospital, Kaohsiung Medical University, Kaohsiung, Taiwan; 11 Department of Biomedical Engineering, National Cheng Kung University, No.1 University Road, Tainan City, Taiwan; Queen Mary University of London, UNITED KINGDOM

## Abstract

Vertical vibration (VV) is a whole-body vibration with mechanical loading that commonly used in rehabilitation and sports training to increase athlete muscle strength. Our previous study showed that low-magnitude, low-frequency VV at 8 Hz and 10 Hz increased myoblast myogenesis. Herein, we investigated whether a VV frequency at low-frequency 5–10 Hz has anabolic effects on tenocytes and improves tendon stiffness. In primary tenocytes, 10 Hz VV treatment increased the tenogenic marker gene expression of tenomodulin and extracellular matrix type I collagen but decreased decorin expression. qPCR and Enzyme-Linked Immunosorbent Assay (ELISA) results showed that TGF-β1 expression was increased in tenocytes after 3 days of 10 Hz VV treatment in vitro and in Achilles tendons after 3 weeks in vivo. Tenomodulin expression and Achilles tendon stiffness were significantly increased in Achilles tendons after 3 weeks in vivo. We also showed that the TGF-β1 receptor inhibitor SB431542 (10 μM) decreased the expression of tenomodulin and type I collagen but increased the decorin expression in tenocytes. These results indicated that the 10 Hz VV stimulated anabolic effects in tenocytes by increasing TGF-β1 expression that subsequently increases the expression of tenomodulin and type I collagen, and increased the Achilles tendon stiffness. This study provides insight into the low-frequency 10 Hz VV treatment improves tendon properties and can minimizes the risk of ligament/tendon reinjure during rehabilitation.

## Introduction

Whole-body vibration (WBV) is a mechanical loading and oscillation [1, 2] therapy that is broadly used in rehabilitation facilities to enhance the performance of frail, institutionalized patients [3] and in sports training to increase the muscle strength of athletes [4, 5]. There are a variety of methods for delivering WBV to the body, such as via a seesaw or vertical vibration (VV) platform [6]. It has been demonstrated to enhance performance in both untrained [7] and athletic [4] young adults, healthy elderly people [5], and frail institutionalized patients [3]. The effect of VV on bone and muscle has been extensively studied [8], but relatively few studies have been reported regarding its effect on tendon. The reported optimal VV frequency for tendon adaptation varies among studies, and most studies use low-magnitude (<1 G; G = 9.98 m/s^2^), high-frequency (less than 20–80 Hz) VV treatment. Hansson et al. (1988) observed a significant increase in IGF-I immunoreactivity in the rat tibia anterior tendon in response to 4-hour-long vibration trauma (81 Hz) during 2 consecutive days [9]. Legerlotz et al. (2007) showed that a 2 G, 25 Hz VV treatment for 12 weeks had no effect on either the mechanical properties or the cross-sectional area of rat Achilles tendons [10]. VV with a frequency of 30 Hz increases the cross-sectional area of tendon but did not affect the stiffness of intact tendons and failed to accelerate tendon healing in rats [11, 12]. Low-magnitude, low-frequency VV (less than 20 Hz) may prevent vibration trauma and tendon injury during rehabilitation. It remains unclear whether a lower VV frequency, such as 5, 8 or 10 Hz, would have an anabolic effect on tenocytes. Our previous study showed that low-magnitude, low-frequency VV at 8–10 Hz increased the myoblast myogenesis for 3 days and myotube formation for 9 days [13]. It is suggested that low-frequency VV may also have effects on tenocytes, however, the anabolic effect of low-frequency VV (5–10 Hz) on tenocytes remains undefined.

Tendons are composed of tenocytes and mostly dense collagen fibers. Tenocytes are key for the regulation of physiological homeostasis and pathological derangements of the tendon matrix [14]. Type III collagen, comprising less than 10% of normal tendons, is important in the healing process due to its ability to rapidly crosslink and to stabilize the injured tendon; it is later replaced by type I collagen [15]. Type I collagen comprises more than 90% of normal tendons [16], and it is known to be upregulated by acute exercise [17] and increased during the remodeling phase of healing tendons [18]. Promoting type I collagen production can increase tendon strength and tendon healing [19]. Tenomodulin, tenascin and decorin are tenogenic differentiation markers and play critical roles in tendon tissue formation [20–23]. Moreover, the transforming growth factor-beta (TGF-β) family is critical for tendon adaptation during exercise and tendon healing. During development, TGF-β1 is involved in hematopoiesis and endothelial differentiation; TGF-β2 affects cardiac, lung, craniofacial, limb, eye, ear, and urogenital system development; and TGF-β3 influences pathogenesis and pulmonary development [24].

The mechanical properties of tendons are thought to be affected by different loading levels. Tendons transmit force from muscle to bone and provide range of motion to joints [25]. Changes in the mechanical properties of tendons, such as stiffness, have been reported to influence the risk of tendon injuries in athletes [26] and the elderly [27], thereby affecting motor function execution. In a human study, unloading resulted in reduced gastrocnemius tendon stiffness, and resistance exercise attenuated this condition [28]. In a rabbit model, stress shielding rabbit patellar tendons altered the mechanical properties of collagen fascicles [29].

The effective frequency of VV varies among studies. We have previously shown that VV treatment at frequencies of 8 and 10 Hz increases the myogenesis of C2C12 myoblasts [13]. Herein, we investigated whether low-magnitude, low-frequency (5 Hz, 8 Hz and 10 Hz) VV treatment induced an anabolic effect on tenocytes. We demonstrated that 10 Hz VV stimulated the anabolic effect of tenocytes and the stiffness of intact Achilles tendons by increasing the expression of TGF-β1, which was followed by an increased expression of tenomodulin and type I collagen that then improved Achilles tendon stiffness via mechanotransduction.

## Materials and methods

### Tenocyte isolation

Tenocytes were isolated from the Achilles tendons of 10-month-old domestic pigs (N = 6; Animal Technology Institute, Taiwan) by digesting the tissue with type I collagenase for 24 hours at 37°C. The procedure for the euthanasia of the pigs was using an anaesthetic overdose of 20 mg of sodium thiopental per kilogram of weight, administered via the vena cava, and animal experiments were performed with the approval of the Kaohsiung Medical University Animal Care and Use Committee. Tenocytes were maintained in Dulbecco's modified Eagle’s medium-nutrient mixture F-12 medium (DMEM-F12; Life Technologies, California, USA) containing 10% fetal bovine serum (FBS; Gibco BRL, Carlsbad, CA, USA) in a humidified atmosphere of 5% CO_2_ at 37°C. The cells were used before their seventh passage.

### VV treatment of tenocytes

**Fig 1:**
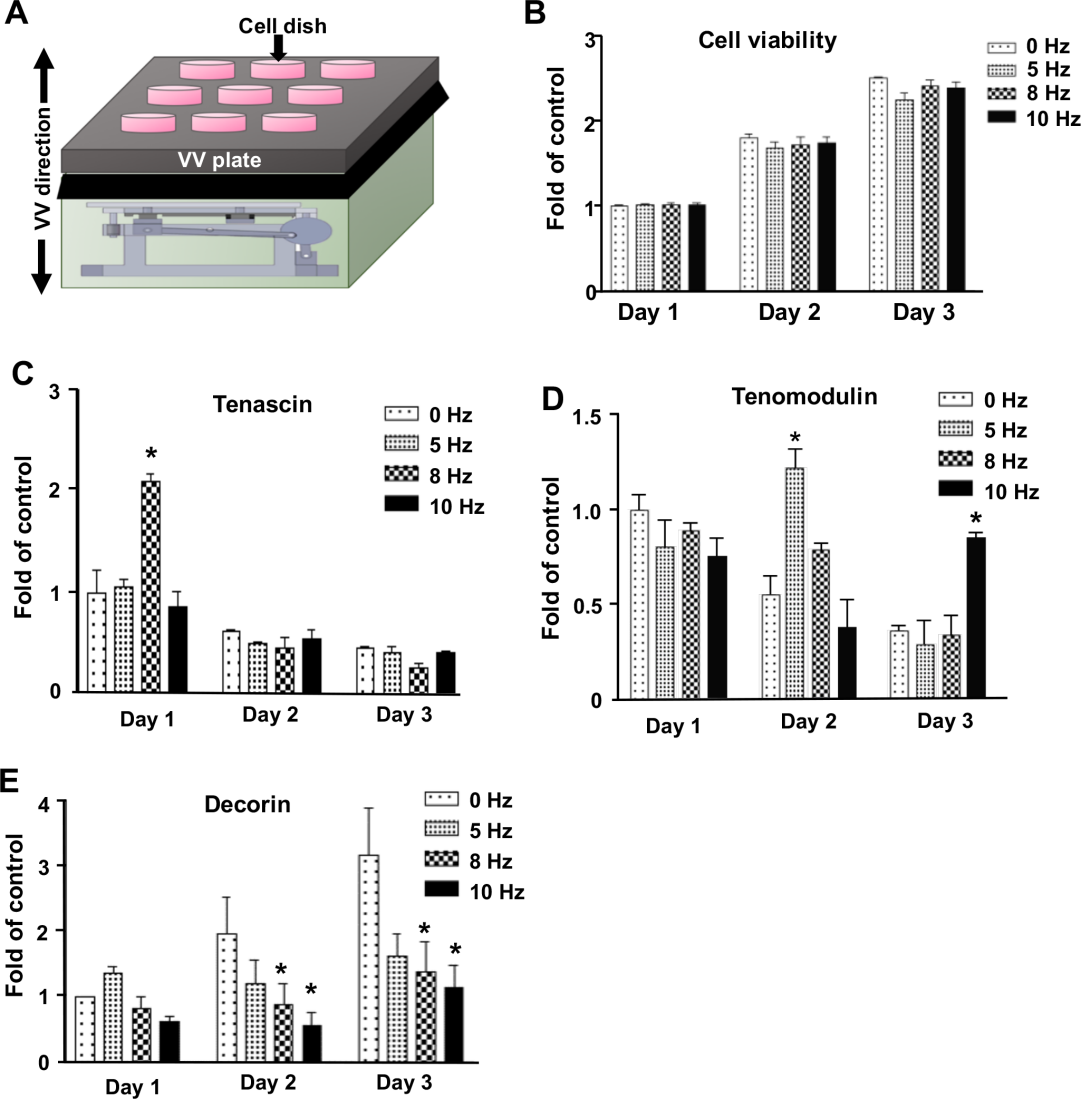
Vertical vibration (VV) treatment increases the expression of the tenogenic marker gene tenomodulin in primary tenocytes. (A) Schematic diagram of VV treatment of tenocytes by using VV device. Arrow indicates the oscillating direction of VV. (B) Quantification of cell viability using the MTT assay in each VV-treated group (5, 8 and 10 Hz); results are expressed as ratios of the treated cells to the control cells (0 Hz), for which the ratio was defined as 1. Determination of tenogenic gene expression, including (C) *Tenascin*, (D) *Tenomodulin and* (E) *Decorin*, by using qPCR analysis. Results are expressed as ratios of the treated cells to the control cells (0 Hz) at day 1, for which the ratio was defined as 1. * P<0.05 for VV-treated versus control cells (0 Hz) at the same day.

To evaluate the effect of VV stimulation on tenocytes, tenocytes (6 x 10^3^ cells/cm^2^) were subjected to vibration stimulation generated by a vertical platform (BodyGreen, Changhua, Taiwan) as described previously [13] (Fig 1A). The control group (0 Hz) were provided without mechanical stimulation to cells for a total 12-min per day for 3 days. The VV treated groups were provided with a frequency of 5, 8 or 10 Hz and an amplitude of 2.0 mm (with a peak-to-peak acceleration of 0.9 G; G = 9.98 m/s2) vibration stimulation to the cells, which a total 12-min intermittent vibration cycles, 90 sec vibration phase and a 30 sec rest phase, per day for 3 days. Cells were collected immediately after the last VV session for further investigation.

### MTT assay for cell viability

The MTT assay was used to evaluate the viability of VV-treated cells that based on the cleavage of a yellow tetrazolium salt (thiazol blue tetrazolium bromide) by metabolically active cells and produces insoluble purple formazan. Briefly, cells were seeded into 24-well plates and were washed with phosphate-buffered saline (PBS); then, 200 μl of MTT solution (0.5 mg/ml in PBS at pH 7.2) was added to each well. After an incubation period of 4 h, the MTT solution was removed, the cells were lysed, and the formazan crystals were dissolved by the addition of 200 μl of dimethyl sulfoxide (DMSO) to each well and incubation at 37°C for 5 min. The optical density (OD) of the solubilized formazan in each well was quantified spectrophotometrically using an ELISA reader (Tecan Sunrise, TECAN Deutschland GmbH, Crailsheim, Germany) at a wavelength of 570 nm.

### Enzyme-Linked Immunosorbent Assay (ELISA)

After VV treatment, 100 μl of the tenocyte culture medium was transferred to a 96-well ELISA plate that had been precoated with mouse anti-TGF-β1 antibody (R&D Systems, Minneapolis, MN), and the plate was incubated for 2 h at room temperature. After the wells had been washed 3 times with PBST, biotinylated anti-TGF-β1 antibody (R&D Systems) was added to each well, and the plate was incubated for 2 h at room temperature. After being washed to remove any unbound antibody-enzyme reagent, streptavidin horseradish peroxidase was added, and the plate was incubated for 20 min at room temperature in the dark. After washing, the substrate solution (a 1:1 mixture of H_2_O_2_ and tetramethylbenzidine) was added for color development, and then, the reaction was terminated by the addition of 2 N H_2_SO_4_. The absorbance of the plate was measured with a microplate reader at 450 nm (Dynex Technologies).

### Total collagen synthesis measurement

Sirius Red dye (Direct Red; Sigma-Aldrich, USA) was used to stain total collagen. At the indicated time intervals, the cells were collected and were lysed using the freeze–thaw method. The cell extracts (50 μl/well) were placed in 96-well plates and were kept in a dry incubator (at 37°C) for desiccation. Each well was washed for 1 min with 200 μl of distilled H_2_O, and this was repeated twice. One hundred microliters of 0.1% Sirius Red stain (0.05 g Sirius Red powder per 50 ml picric acid) was added to each well, and the plate was incubated at room temperature for 1 h. Then, unbound stain was removed by five washes with 200 μl of 0.1 M HCl. The bound stain was extracted with 200 μl of 0.1 M NaOH (5 min) and was mixed well. The extracted stain was placed into a second plate to measure the absorbance at 540 nm (Sirius Red) and 260 nm (DNA content) using a microplate reader.

### Inhibition of transforming growth factor-beta 1 (TGF-β1) signaling in tenocytes

To investigate the role of TGF-β1 signaling in VV-treated tenocytes, SB431542 (10 μM), a specific ATP-mimetic inhibitor of TGF-β1 receptors [30], was used in VV-treated tenocytes. SB-431542 was purchased from STEMCELL Technologies Inc. (Vancouver, BC, Canada). For inhibition of TGF-β1 signaling [30], a stock concentration of 10 mmol/L SB-431542 in DMSO was prepared and then diluted to the final concentration of 10 μM in cell cultured medium for subsequent experiments.

### VV treatment of rats

To evaluate the immediate effect of VV on the Achilles tendon in vivo, the VV of Sprague Dawley (SD) rats was performed. 10-week-old male SD rats were purchased from the National Laboratory Center, and the in vivo experiments were performed with the approval of the Kaohsiung Medical University Animal Care and Use Committee and were housed under a constant temperature and controlled illumination. Rats were randomly divided into two groups (n = 18/group): control group (0 Hz) and 10 Hz treated group (10 Hz). Rats were hung by a belt, and their hind limbs were placed on the VV platform (BodyGreen, Taiwan). The control group (0 Hz) were provided without vibration stimulation to the rat hind limbs for a total 12-min per day for 3 weeks. The 10 Hz treated group were provided with a frequency of 10 Hz and an amplitude of 2.0 mm (0.9 G) vibration stimulation to the rat hind limbs, which a total 12-min intermittent vibration cycles, 90 sec vibration phase and a 30 sec rest phase, per day for 3 weeks. After 3 weeks, the rats were anesthetized with dexmedetomidine injections (0.5 mg/kg, Dexdomitor; Orion Pharma, Esbo, Finland) and ketamine (75 mg/kg, Ketaminol; Intervet, Boxmeer, Holland) and were sacrificed by an overdose of pentobarbital sodium (APL, Stockholm, Sweden). The rat hindlimbs (n = 6/group) were prepared for mechanical property analysis. The rat Achilles tendons were harvested for quantitative real-time PCR (qPCR) (n = 6/group) analysis and paraffin-embedding for immunofluorescence staining (n = 6/group).

### Quantitative real-time PCR

Total RNA was isolated from cells and rat Achilles tendons by using TRIzol reagent (Gibco BRL). The reverse transcription of RNA into cDNA was performed using oligo(dT) primers and the Moloney murine leukemia virus reverse transcriptase. Quantitative real-time PCR (qPCR) was performed in a Bio-Rad iQ5 real-time PCR detection system (Bio-Rad Laboratories Inc., Hercules, CA, USA) using the iQ SYBR Green Supermix (Bio-Rad). Reactions were performed in a 25-μl total volume, which contained cDNA, specific primers for each gene and iQ SYBR Green Supermix. The primer sequences used are shown in Table 1. Specific PCR products were detected by measuring the fluorescence of SYBR Green, a dye that binds to double-stranded DNA [31]. After real-time PCR, a dissociation (melting) curve was generated to check the specificity of the PCR reaction. The relative mRNA expression level was calculated from the threshold cycle (*C*_t_) value of each PCR product and was normalized to that of β-actin by using the comparative *C*_t_ method [32]. All real-time PCR experiments were performed in triplicate and were repeated three times.

**Table 1:** Primer sequences for quantitative real-time PCR.

***Rat's primer***	***Gene sym***	***Forward***	***Reverse***
**Tenocyte marker gene**	*Tenascin*	CCTTTGATGTCACCGGAGTT	GGCGAAAACCCTCTATAGCC
	*Tenomodulin*	GTTTTGGGGGAGCAAACACT	ATGTTTCATCGGTGCCATTT
	*Decorin*	GATCAGCCCAGAGGCATTTA	CCGCCCAGTTCTATGACAAT
	*TypeICollagen*	AAGACATCCCACCAGTCACC	CAGTTCTTGATTTCGTCGCA
	*TypeIIICollagen*	ATAGACCTCAAGGCCCCAAG	CCTCCGACTCCAGACTTGAC
	*TGF-β1*	GGCCAGATCCTGTCCAAACT	GGGTGACTTCTTTGGCGTAG
	*TGF-β2*	GCAAGTGGGAGAGGAAGAGA	TTCCTCCAAGCTTGCACTTT
	*TGF-β3*	CTCTCTGTCCACTTGCACCA	GCATCTCTTCCAGCAACTCC
	*β-actin*	CCACCCGAGGAGGGCAG	GGCTGCCCACTCAAAATAAACC
***Porsine's primer***	***Gene sym***	***Forward***	***Reverse***
**Tenocyte marker gene**	*Tenascin*	TCCCAGTGCTCAGTGGATCT	CCTCCAGTCTGCTCAGAAGC
	*Tenomodulin*	TCGCCCTAACGCTAATTGTC	TTTCATCAGTGCCATTTCCA
	*Decorin*	ACCGCTTTCCTGAAGTTCCT	TTGTTTTGCAGATCCAGCAG
	*TypeICollagen*	CCAAGAAGAAGGCCAACAAG	ACGTCATCGCACAACACATT
	*TypeIIICollagen*	TCCTGGTATTCCTGGGAGAA	CCTGCTACTCCAGCCTTGAC
	*TGF-β1*	CACCCCAGATCCTCCTACCT	GTCAGCACTAGCAGCCACAG
	*TGF-β2*	TCGTCGCTCCAAGAAAACTT	TCGAGAGTCAATGTCCGAAA
	*TGF-β3*	GCAAACTCAGGCTCACCAGT	CCCTGGATCATGTCGAATTT
	*β-actin*	AGATCTGGCACCACACCTTC	CATACATGGCAGGGACATTG

### Immunofluorescence

Cells on coverslips were washed thrice with PBS and then were fixed with 4% paraformaldehyde in PBS for 20 min at room temperature. After washing 3 times with PBS, the samples were permeabilized with 0.5% Triton X-100 in PBS for 10 min, rinsed with PBS, and then immunostained with anti-collagen type I antibody (Millipore; Billerica, MA, USA) overnight at 4°C. After washing 3 times with PBS, the coverslips were exposed to Alexa Fluor 594-labeled secondary antibodies (Molecular Probes, Inc., Eugene, OR, USA) for 1 h; then, the nuclei were stained with 4,6-diamidino-2-phenylindole (DAPI; Sigma-Aldrich Co., St. Louis, MO, USA). The coverslips were mounted with anti-fade solution (Molecular Probes). Images of samples from independent experiments were captured using a fluorescence microscope. A total of 6 representative images per sample were scanned with a 400 times magnification in a total of 1760 digital images in TIFF file extension format, and the stain intensity of scanned images were analyzed to quantify the total amount of collagen type I using Image-Pro Plus software (Media Cybernetics, Silver Spring, MD, USA). For each sample, staining without primary antibody was performed with a side-by-side parallel specimen as a negative control; these stains all yielded blank images.

### Immunohistochemistry (IHC)

SD rat tendon were fixed and paraffin-embedded as described previously [33]. IHC was performed using the ImmunoCruz Staining System (Santa Cruz Biotechnology, Inc. Dallas, Texas). Sections were incubated in 0.1% EDTA for 10 min at 100°C for antigen retrieval [33, 34]. After incubating with 5% BSA/PBS (Sigma, Saint Louis, MO, USA) blocking solution for 2 hr at room temperature, sections were labeled with anti-TGF-β1 antibody (R&D Systems; Minneapolis, MN, USA) overnight at 4°C in a humid chamber. After washing with PBS, sections were incubated with a biotinylated secondary antibody (Dako, Carpinteria, CA) for 1 hr and then incubated with horseradish peroxidase-conjugated streptavidin (Dako, Carpinteria, CA) for 1 hr. The reaction was developed using a 3,3’-diaminobenzidine solution containing 0.01% hydrogen peroxide, resulting in a brown color [33]. Sections were then counterstained with hematoxylin. Images were taken using a microscope equipped with a digital CCD camera (Eclipse 50i; Nikon Inc., MI, USA). For quantification, the sections of TGF-β1 staining were digitalized at 400 times magnification in a total of 2560*1920 pixel and 300 dpi digital images using TissueFAXS microscope (TissueGnostics GmbH, Vienna, Austria), and the digital images in JPG file format were analyzed to quantify the total amount of TGF-β1 staining using HistoQuest (TissueGnostics, Los Angeles, CA) analysis software. Total area of TGF-β1 staining was detected after automatic color separation by HistoQuest. The staining intensity was measured as mean intensity of all pixels and the HistoQuest automatic background threshold range of values is from 5 to 255.

### Evaluation the mechanical properties of rat Achilles tendons

The determination procedure was modified from the previous study[35]. After euthanasia, the hindlimbs were transected at the knee joints. The hindlimbs were soaked with phosphate buffer solution and stored in -20°Cbefore mechanical testing. Twenty minutes before testing, specimens were thawed at 37°C by water bath. The gap between tendon ends was measured after the surrounding connective tissues were dissected from the Achilles tendon-calcaneus complex. The tendon-calcaneus complex was isolated, and the length, width, and thickness of the tendon were measured by using dial calipers (Mitotoyo, Tokyo, Japan). Cross-sectional area was calculated as the product of mean width and thickness. The two ends of tendon-calcaneus complex were secured with tissue clamps. Mechanical properties of the Achilles tendon were determined by the Microforce Testing System (MTS, Tytron 250, MTS Systems Corp., Eden Prairie, Minnesota). A constant stretching rate (10 mm/min) was applied to the specimens until separation of the tissues. Force-displacement curves of the tested tendons were constructed. The maximum load, ultimate stress and stiffness were calculated by force-displacement curve, where ultimate stress was the maximum breaking force and stiffness was calculated from the slope of linear range on force-displacement curve.

### Statistical analysis

Each value represents the means ± SD of three independent experiments. One-way ANOVA and Paired samples t-test statistical analyses were used to compared the difference at different time groups of each experiment. The multiple comparisons with significance were further verified by using Tukey’s Test (Studentized Range Distribution) of post-hoc test to avoid type I error. Statistical significance was set at P < 0.05.

## Results

### VV treatment enhance the tendonogenic marker genes expression of tenomodulin in primary tenocytes

We investigated the anabolic effects of VV treatment on primary tenocytes. Our previous study showed that low-magnitude VV at 8–10 Hz for 3 days is enough for in vitro evaluation of the anabolic effect of VV on myoblasts [13], therefore, we evaluated the effect of VV stimulation on at 5–10 Hz for 3 days on tenocytes. The cell viability results showed that VV (5, 8,10 Hz) treatment in 3 days in vitro did not influence the cell viability of tenocytes compared to control cells (0 Hz) (Fig 1B). We evaluated whether the VV treatment (5, 8,10 Hz) altered the gene expression of tendonogenic marker genes, such as the tenomodulin, tenascin, and decorin etc., compared to control cells (0 Hz) in tenocytes by using qPCR. We showed that the gene expression of tenascin increased significantly in 8 Hz VV at day 1, but showed no difference on tenascin expression at day 2 and day 3 compared to control cells (Fig 1C). VV treatment showed dynamic anabolic tenomodulin expression with significant increase tenomodulin expression at day 2 after 8 Hz VV and at day 3 after10 Hz VV treatment compared to control cells (0 Hz) (Fig 1D). By contrast, the gene expression of decorin showed significant decrease at day 2 and day 3 of 8 Hz VV and 10 Hz VV treatment in a dose-dependent manner compared to control cells (0 Hz) (Fig 1E).

#### The expression of type I and type III collagen in VV-stimulated tenocytes

**Fig 2:**
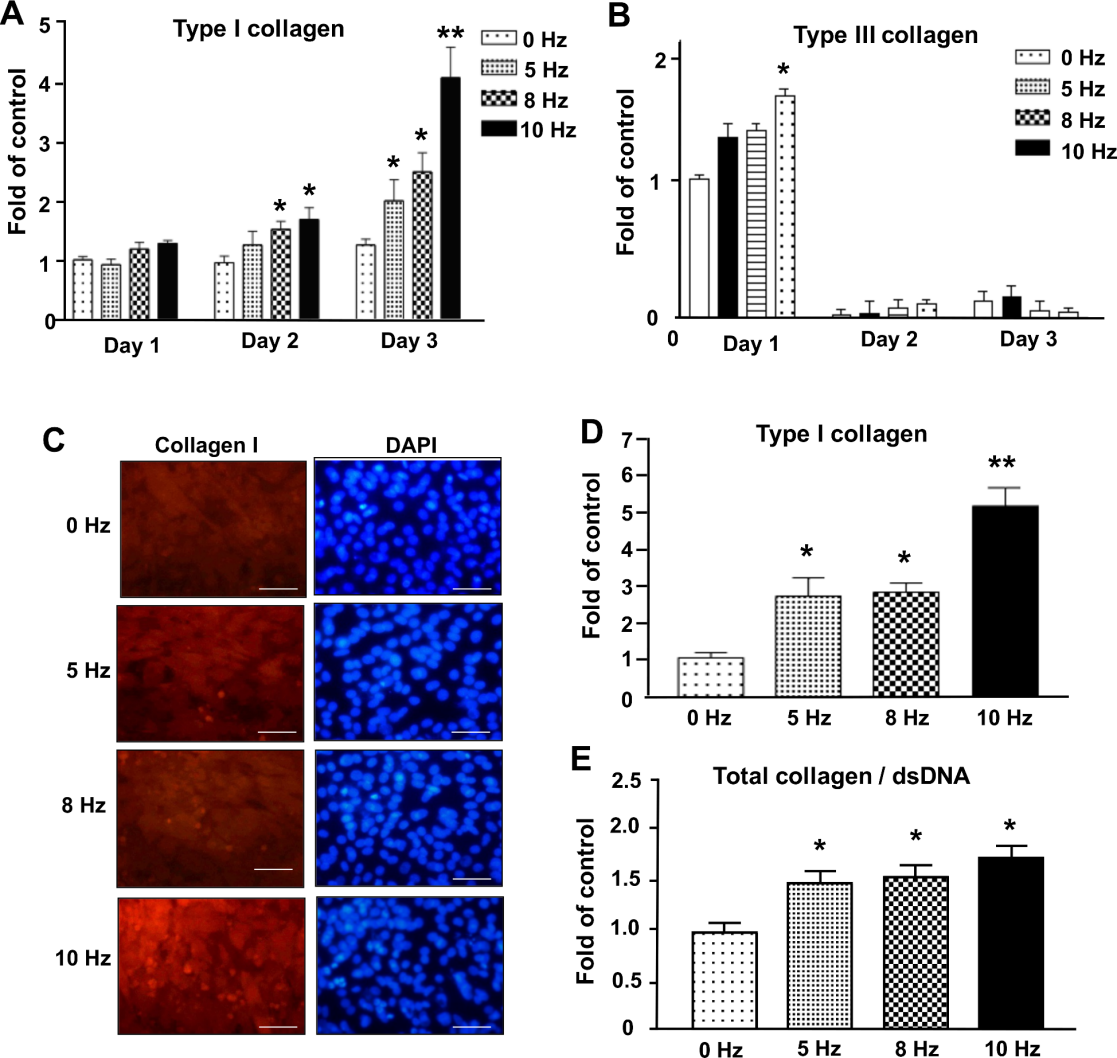
Detection of the expression of type I and type III collagen in VV-stimulated tenocytes. The gene expression of (A) *Type I collagen* and (B) *Type III collagen*, as determined by using qPCR analysis. Results are expressed as ratios of the treated cells to the control cells (0 Hz) at day 1, for which the ratio was defined as 1. *P<0.05, **P<0.01 for VV-treated versus control cells (0 Hz) at the same day. (C) Immunofluorescence results of type I collagen in tenocytes after VV treatment. Red: type I collagen; blue: DAPI, nuclear counterstain. Bar: 60 μm. (D) Quantitative results of type I collagen immunofluorescence. (E) The normalized result of total collagen expression/dsDNA in VV-treated tenocytes. *P<0.05, **P<0.01 for VV-treated versus control cells (0 Hz).

The tenogenic marker genes type I and III collagen expression were detected to represent the tenogenic activation. We found that type I collagen significantly increased at day 2 after 8 Hz and 10 Hz VV treatment, and then increased dose dependently after VV treatment at day 3 and had a 4-fold increase after 10 Hz VV treatment (Fig 2A) compared to control cells (0 Hz). However, the expression of type III collagen only exhibited a significant increase with 10 Hz VV-treatment for day 1, and there is no significant difference in day 2 and 3 (Fig 2B) compared to control cells (0 Hz). These results suggested that type I collagen might be an important tendonogenic extracellular matrix contributing to the VV effect on tenocytes. Therefore, we detected the expression of type I collagen in tenocytes after VV treatment for 3 days by using immunofluorescence analysis for type I collagen staining and Sirius Red staining for total collagen determination. The immunofluorescence images showed that the expression of type I collagen increased after VV stimulation for 3 days compared to control cells (0 Hz), especially that of 10 Hz VV treatment (Fig 2C). Consistent with the qPCR results, the quantitative results of type I collagen staining showed a significant increase of the expression of type I collagen in a dose-dependent manner after VV treatment, which had a 2-fold increase after 5 Hz and 8 Hz VV treatment and 5-fold increase after 10 Hz VV treatment compared to control cells (0 Hz) (Fig 2D). The quantitative results of Sirius Red staining also showed a significant increase of the total collagen expression in a dose-dependent manner after VV treatment compared to control cells (0 Hz) (Fig 2E).

#### The effect of 10 HZ VV treatment on intact rats’ Achilles tendon

**Fig 3:**
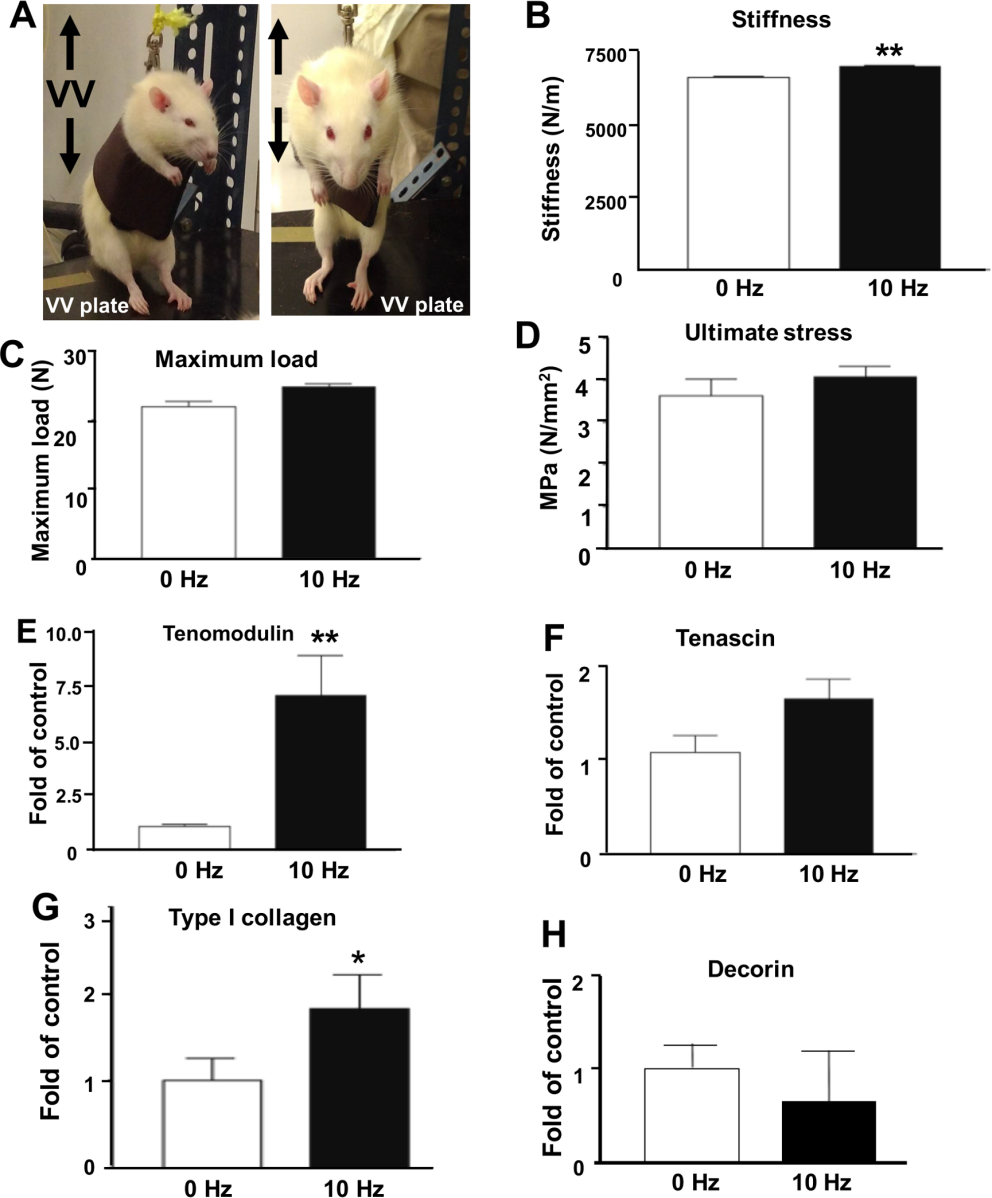
The effect of 10 Hz VV treatment on the mechanical properties and tenogenic gene expression of rat Achilles tendons. **(A)** Photographs show the rats positioned on the VV platform (VV plate) for intermittent vibration stimulation. Arrow indicates the oscillating direction of VV. The mechanical properties of tendon including **(B)** stiffness, **(C)** maximum load and **(D)** ultimate stress were measured after 3 weeks of 10 Hz VV treatment. The tenogenic marker gene expression levels, including **(E)** *Tenomodulin*, **(F)** *Tenascin*, **(G)** *Type I collagen* and **(H)** *Decorin*, in rat Achilles tendons after 10 Hz VV treatment were determined by using qPCR. *P<0.*05*, **P<0.*01* for 10 Hz VV-treated versus control cells (0 Hz).

The Achilles tendon is one of the most important tendons of the human body and 30–50% of sports-related injuries are involved in Achilles tendon injuries. We found that VV treatment, especially 10 HZ VV treatment, could increase the expression of important tendonogenic genes such as tenomodulin and type I collagen in vitro. Therefore, we confirmed the effect of 10 HZ VV stimulation on the hindlimb of rat Achilles tendon in vivo (Fig 3A). To measure the effective time for 10 Hz VV on enhancing the mechanical properties of rat Achilles tendon, we first measured the stiffness, maximum load and ultimate stress value. We found that After 10 Hz VV treatment for 3 weeks, the rat Achilles tendon showed significantly increased on stiffness (Fig 3B) with significant increase of the maximum load (Fig 3C) and ultimate stress (Fig 3D) compared to control cells (0 Hz). Since we found that continuously 3 weeks 10 Hz VV treatment can enhance of stiffness of Achilles tendon, we next investigated the anabolic effect of 10 Hz VV treatment on rat Achilles tendon for 3 weeks. The tenogenic gene expression, such as tenomodulin, tenascin, and tendon matrix genes type I collagen and decorin were determined by qPCR. It was found that tenomodulin was significantly up-regulated over 7 folds than control group (0 Hz) in rats’ tendon after 10 Hz VV treatment (Fig 3E), which had the similar regulation of tenomodulin in day 3 VV treatment in tenocytes in vitro. However, the expression of tenascin showed only slightly increase compared to control cells (0 Hz) (Fig 3F). Moreover, a significant increase of type I collagen gene expression was observed in rats Achilles tendon after VV treatment (Fig 3G). The in vivo results showed that a slight decrease without significance of decorin gene expression (Fig 3H) were observed in 10 Hz VV-treated rat Achilles tendon at 3 weeks.

#### VV treatment enhance the expression of TGF-β1 in tenocytes and Achilles tendon

**Fig 4:**
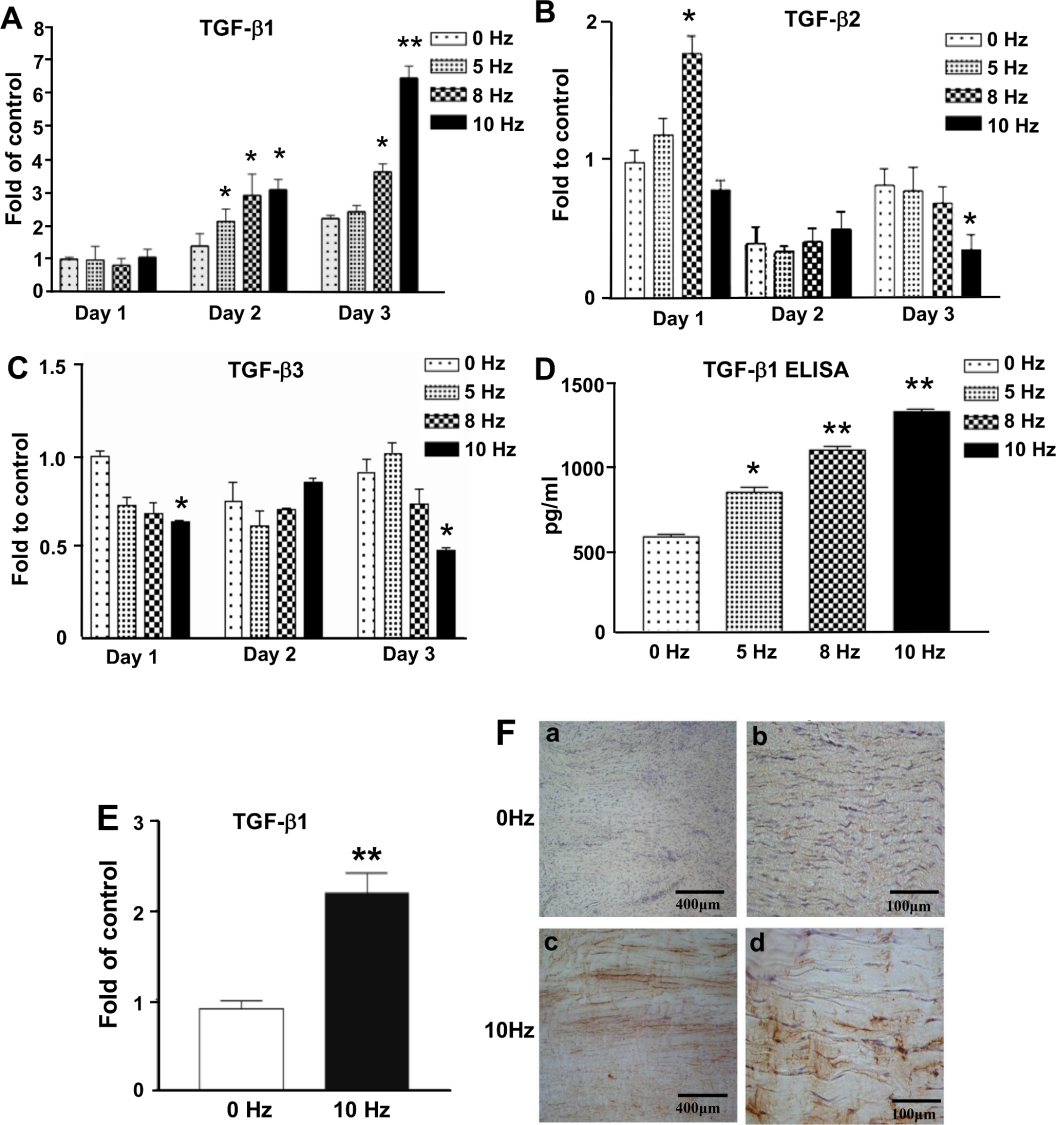
VV treatment increases the expression of TGF-β1 in tenocytes and rat Achilles tendons. Gene expression of TGF-β isoforms in VV-treated tenocytes, including (A) *TGF-β1*, (B) *TGF-β2* and (C) *TGF-β3*, were measured by using qPCR. Results are expressed as ratios of the treated cells to the control cells (0 Hz) at day 1, for which the ratio was defined as 1. * P<0.05 and ** P<0.01 for VV-treated versus control cells (0 Hz) at the same day. (D) The protein expression of TGF-β1 in tenocytes was determined by ELISA analysis. * P<0.05 and ** P<0.01 for VV-treated versus control cells (0 Hz). (E) The in vivo effect of 10 Hz VV stimulation on *TGF-β1* gene expression, as determined by qPCR. ** P<0.01 for 10 Hz VV-treated versus control cells (0 Hz). (F) Immunofluorescence staining of TGF-β1 in rat Achilles tendons after VV stimulation. Brown: TGF-β1; blue: hematoxylin counterstain. Bar: 400 μm.

TGF-β family were central for tendon adaptation by promoting type I collagen synthesis [36]. To understand whether VV treatment influence the expression of TGF-β family, we investigated the dose-dependent effect VV stimulation on the regulation of TGF-β1, TGF-β2 and TGF-β3 in tenocytes by using qPCR. The gene expression of TGF-β1 showed dose dependently increased at day 2 (P<0.05) after VV treatment, and then increased at day 3 after 8 Hz VV treatment (P<0.05) and had a 4-fold increase after 10 Hz VV treatment (P<0.01) (Fig 4A). The gene expression of TGF-β2 showed significant increase only at day 1 after 8 Hz VV (P<0.05) treatment, and then decreased at day 3 after 10 Hz VV (P<0.05) treatment (Fig 4B). The gene expression of TGF-β3 showed significant decrease at day 1 and day 3 (P<0.05) after 10Hz VV treatment (Fig 4C). These results suggested TGF-β1 might be the most important TGF-β isoform contributing to the VV effect on tenocytes. Therefore, we detected the TGF-β1 protein expression in VV-treated tenocytes by using ELISA analysis. In agreement with the qPCR results, the ELISA results showed that the protein expression of TGF-β1 significantly increased in VV-treated tenocytes in a dose-dependent manner with the highest TGF-β1 expression at10 Hz VV treatment (Fig 4D). We also investigated the in vivo effect of 10 Hz VV treatment on the expression of TGF-β1 in rat Achilles tendon for 3 weeks by using qPCR and IHC staining. The qPCR results showed that the gene expression of TGF-β1 was significantly increased in rat Achilles tendon (Fig 4E). IHC staining results showed that the protein expression of TGF-β1 was increased in rat Achilles tendon after 10 Hz VV treatment for 3 weeks (Fig 4F).

#### TGF-β1 signaling inhibitor decreased the expression of tenomodulin and type I collagen, but enhanced the expression of decorin in tenocytes

**Fig 5:**
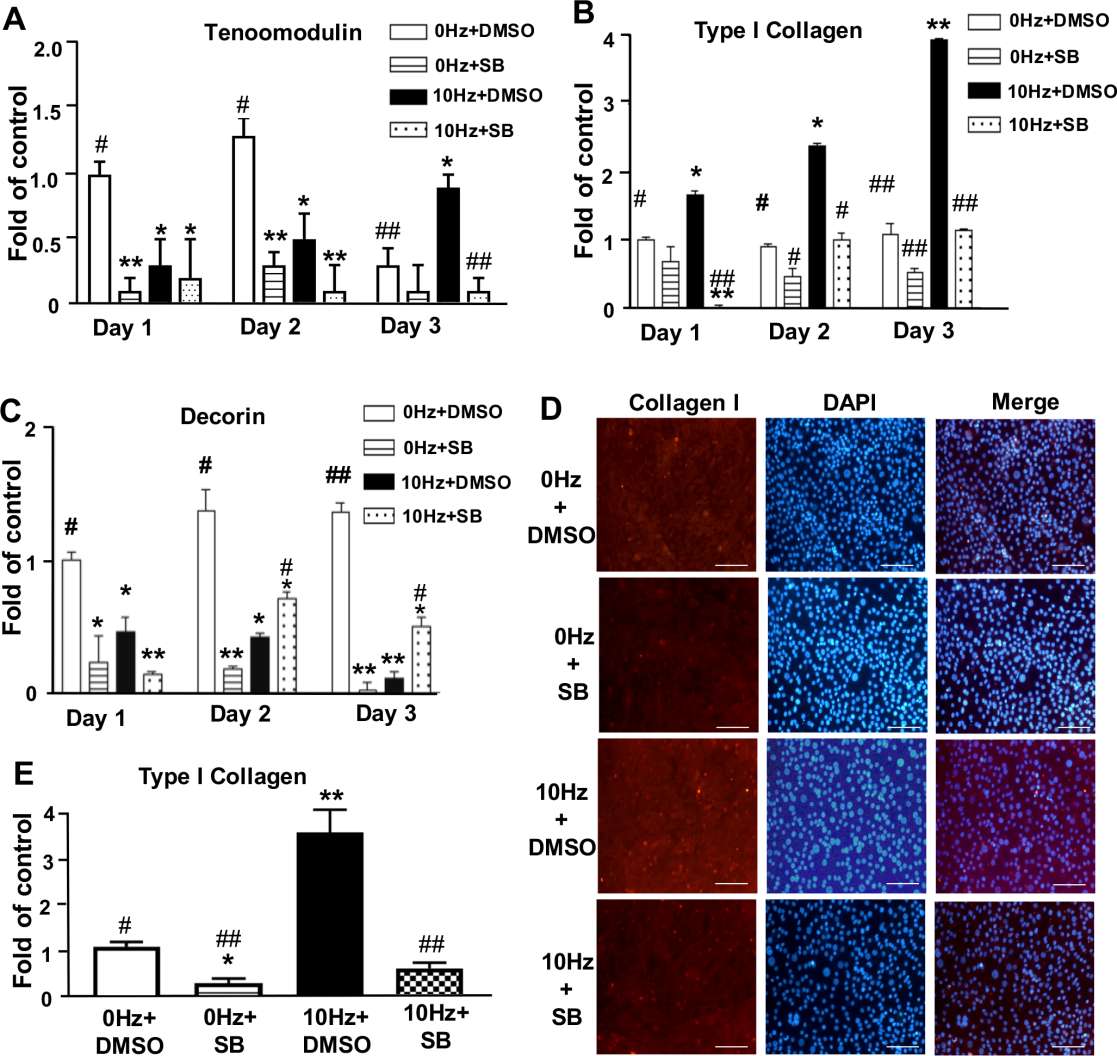
TGF-β1 inhibitor SB431542 decreased the expression of *tenomodulin* and *type I collagen* but increased the expression of *decorin* in tenocytes. Evaluation of the gene expression of **(A)** *Tenomodulin*, **(B)** *Type I collagen* and **(C)** *Decorin* in VV-treated tenocytes by using qPCR in control cells (0 Hz) treatment with vehicle control DMSO (0 HZ+DMSO), control cells (0 Hz) treatment with 10 μM SB431542 (0 HZ+SB), 10 Hz VV-treated cells with DMSO (10 HZ+DMSO) and 10 Hz VV-treated cells with 10 μM SB431542 (10 HZ+SB). Results are expressed as ratios of the treated cells to the control cells (0 Hz) at day 1, for which the ratio was defined as 1. * P<0.05 and ** P<0.01 for VV-treated groups versus 0 HZ+DMSO group at the same day. # P<0.05 and ## P<0.01 for VV-treated groups versus 10 HZ+DMSO group at the same day. (D) Images of IHC stain of type I collagen in in control cells (0 Hz) treatment with vehicle control DMSO (0 HZ+DMSO), control cells (0 Hz) treatment with 10 μM SB431542 (0 HZ+SB), 10 Hz VV-treated cells with DMSO (10 HZ+DMSO) and 10 Hz VV-treated cells with 10 μM SB431542 (10 HZ+SB). Red: type I collagen; blue: DAPI, nuclear counterstain. Bar: 100 μm. (E) Quantitative results of IHC stain of type I collagen. Results are expressed as ratios of the treated cells to the control cells (0 HZ+DMSO), for which the ratio was defined as 1. * P<0.05 and ** P<0.01 for VV-treated groups versus 0 HZ+DMSO group. ## P<0.01 for VV-treated groups versus 10 HZ+DMSO group.

To further investigate the role of TGF-β1 signaling in VV-enhanced tenogenic gene tenomodulin and its relationship with type I collagen and decorin, the specific TGF-β1 receptor inhibitor SB431542 (10 μM) was used in VV-treated tenocytes. The gene expression of tenomodulin, type I collagen and decorin were investigated using qPCR analysis in control cells (0 Hz) treatment with vehicle control DMSO (0 HZ+DMSO), control cells (0 Hz) treatment with 10 μM SB431542 (0 HZ+SB), 10 Hz VV-treated cells with DMSO (10 HZ+DMSO) and 10 Hz VV-treated cells with 10 μM SB431542 (10 HZ+SB). As shown in Fig 5A, we found that the SB431542-treated tenocytes showed significantly decreased the gene expression of tenomodulin in 0Hz+SB group (**, P<0.01) at day 1 and day 2 compared to 0Hz+DMSO group, and that of 10Hz+SB group (##, P<0.01) at day 3 compared to 10Hz+DMSO group, which indicted that TGF-β1 was important for regulating the expression of tenomodulin in tenocytes. In 0Hz+SB group showed slightly decrease without significance in the expression of type I collagen compared to 0Hz+DMSO group, however, in 10Hz+SB group showed that SB431542 exhibited a significant inhibitory effect on type I collagen expression (##, P<0.01) during 3 days VV treatment compared to 10Hz+DMSO group (Fig 5B), which indicted that TGF-β1 can regulate the gene expression of type I collagen. Interestingly, in 0Hz+SB group at day 1(*, P<0.05), day 2 and day 3 (**, P<0.01), the expression of decorin was down-regulated compared to 0Hz+DMSO group. However, in 10Hz+SB group showed that SB431542 exhibited an enhance effect on decorin expression at day2 and day 3 (#, P<0.05) compared to 10Hz+DMSO group (Fig 5C), which indicated that TGF-β1 may have stimulated effect on the decorin expression in control (0Hz) tenocytes and 10 Hz VV-reduced decorin expression was regulated by TGF-β1 in tenocytes. The images of IHC staining (Fig 5D) and quantification of type I collagen (Fig 5E) showed that 10 Hz VV enhanced the staining of type I collagen, and that of staining of type I collagen was suppressed in the SB431542-treated tenocytes.

## Discussion

**Fig 6:**
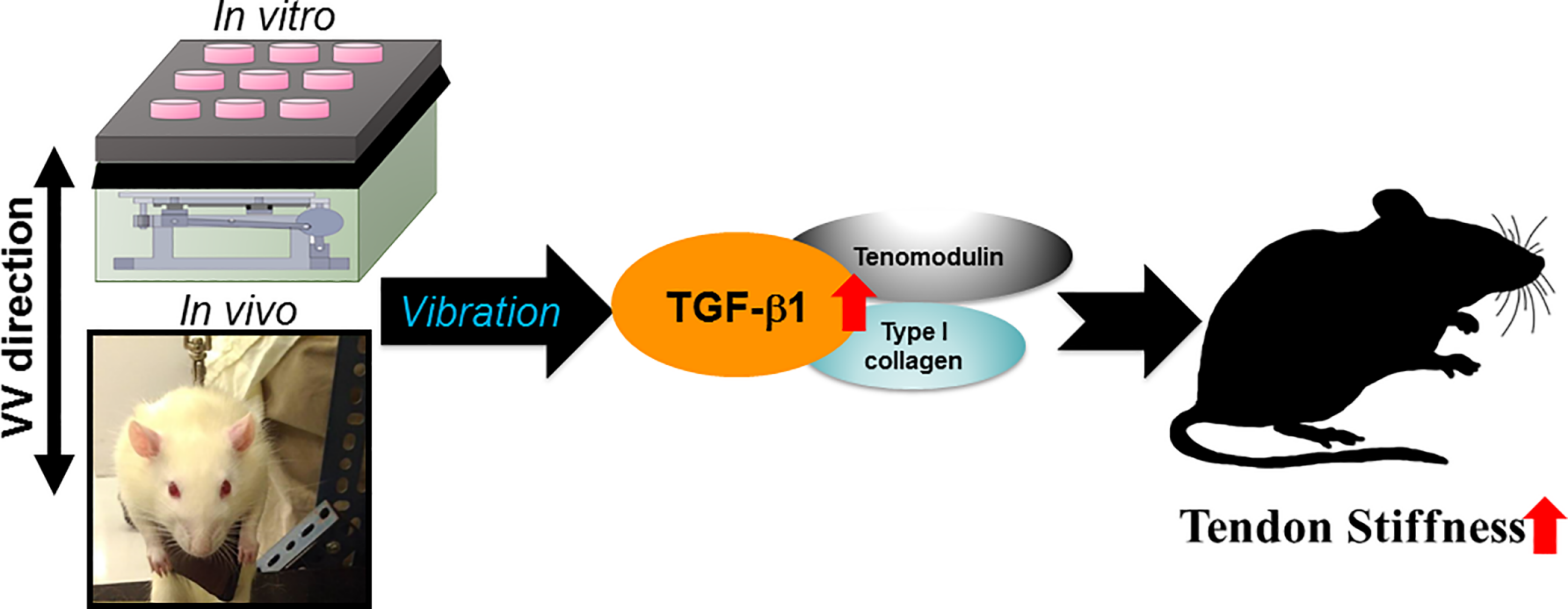
Schematic diagram of TGF-β1 mediates VV enhanced tenomodulin and type I collagen for improving the stiffness of rat Achilles.

In rehabilitation and sports activities, mechanical load is widely used to improve joint flexibility in humans. VV is a kind of mechanical load. VV can activate mechanical stress-responsive cells through the mechanical deformation of the plasma membrane, and this response is advanced by the nucleus and a set of signaling events that cause changes in transcription, translation or cell proliferation [37]. The physiological mechanisms of VV include improved blood flow [38] and enhanced hormone release, such as that of testosterone, which leads to a subsequent decrease in cortisol and growth factor levels [39, 40]. We previously showed that VV at 8–10 Hz for 3 days increased the anabolic effect of myoblast and myogenesis [13]. In this study, we showed that the physiological mechanism of 10 Hz VV was by stimulating anabolic effects via TGF-β1 expression that subsequently increased the expression of the tenogenic genes tenomodulin and extracellular matrix type I collagen, and increased the stiffness of rat Achilles tendons and benefit for improved tendon properties (Fig 6).

Tendon can absorb VV forces; however, high-frequency VV, shearing and mechanical overloading may cause tendon injury [41]. Low-frequency VV reduces the potential for further harm and mechanical overloading of tendon injuries. Relatively few studies have been studied the effect of lower frequency VV (<20 Hz) on the structural stiffness and anabolic effect of Achilles tendons. High-frequency, 30 Hz VV for 30 minutes a day for 12 days fails to quicken the healing of rat medial collateral ligaments and does not affect the structural stiffness of the Achilles tendon [12]. Studies have shown that using treatments of high-frequency 30-Hz sinusoidal vibration daily for 5 days a week for 5 weeks increased the stiffness of intact rat flexor carpi ulnaris tendon but failed to increase maximum loading [11]. In this study, we, for the first time, demonstrate that the 10 Hz VV enhanced TGF-β1 expression, which subsequently increased the tenomodulin and type I collagen expression and increased the stiffness of Achilles tendons of rat.

The physiological mechanisms by which VV increases the expression of TGF-β1, tenomodulin and collagen synthesis in tenocytes have not been investigated. Type I collagen is the most abundant extracellular matrix (ECM) protein in tendons. Increased levels of type I collagen expression are crucial for the tendon response to mechanical loading [42]. Although high-frequency, 30 Hz VV every day for 12 days increased collagen synthesis in the Achilles tendon, this treatment did not affect the structural stiffness of the Achilles tendon, and the mechanisms involved in the collagen synthesis remain unknown [12]. TGF-β1 is upregulated by exercise in intact [42, 43] and healing tendons [44]. Cyclic strain (1 Hz, 10% equibiaxial strain) transiently upregulated the expression of TGF-βs [45]. Cyclical strain (1 Hz, 5% uniaxial strain) modulates metalloprotease and matrix gene expression in human tenocytes via activation of TGF-β1 [46]. TGF-β1 can improve contraction and the mechanical strength of collagen [47]. TGF-β1 is also a potent inducer of type I collagen expression in intact tendons and tenocytes [24, 42, 48]. Studies using pellet cultures to induce chondrogenesis in chick mesodermal cells showed that TGF-βs have an antichondrogenic effect, which is followed by an increase in tenomodulin expression [49]. In this study, we initially showed that the application of 10 Hz VV acted as an anabolic stimulus of tenocytes to promote the expression of TGF-β1, which increased the expression of tenomodulin and type I collagen, improving the mechanical properties of the tendon matrix including the Achilles tendon stiffness. Although we have evaluated the effect of 10 Hz VV treatment on intact Achilles tendons for 3 weeks, but the effect of longer durations of 10 Hz VV treatment on the Achilles tendon needs to be further investigated.

This study demonstrated that the expression of TGF-β1, tenomodulin and type I collagen was increased and that Achilles tendon stiffness was improved following 10 Hz VV stimulation, which may indicate that 10 Hz is the lowest frequency of VV for enhancing the mechanical properties of intact Achilles tendons. Tenomodulin is a well-established gene marker and a mechanosensitive gene in mature tendons [50]. During the mechanotransduction of VV stimulation in tenocytes, the expression of tenomodulin showed no dose-dependent effect during the first 2 days of VV stimulation, but on the third day, the 10 Hz VV treatment significantly increased tenomodulin. In rat Achilles tendons, the 10 Hz VV treatment for 3 weeks increased the expression of tenomodulin. These results suggested that tenomodulin expression is effectively induced only after 3 days of low-frequency 10 Hz VV treatment. Moreover, we suggested that the expression of tenascin and decorin may not be the major factors involved in increasing rat Achilles tendon stiffness following low-frequency 10 Hz VV treatment. We showed that tenascin was upregulated only after the 8 Hz VV treatment for 1 day in tenocytes in vitro, and 10 Hz VV did not affect tenascin expression in the Achilles tendon after 3 weeks VV treatment. Consistent with previous studies, we showed that TGF-β1 suppressed the expression of decorin in tenocytes [44]. Although decorin expression was dose-dependently decreased in tenocytes after 8 and 10 Hz VV treatment for 3 days in vitro, there was only a slight, insignificant decrease in decorin gene expression in rat Achilles tendons after 3 weeks VV treatment. These results suggested that VV treatment may have a transient effect on tenascin and decorin expression in tenocytes during the first 3 days of treatment but without sustained effect on tenocytes after 3 weeks VV treatment.

Activation of TGF-β1 regulated the cyclical strain-induced matrix gene expression in human tenocytes [46]. In this study, we showed that TGF-β1 synthesis was increased in the Achilles tendon. The TGF-β1 receptors inhibitor, SB431542, significantly suppressed the upregulation of tenomodulin and collagen type I expression and the downregulation of decorin expression induced by VV in tenocytes, which indicated that the mechanotransduction of VV stimulation is primarily mediated through TGF-β1 in tenocytes. These results demonstrate that the 10 Hz VV enhanced TGF-β1 expression that subsequently increased the tenomodulin and type I collagen expression. Downstream, an increase in tendon stiffness was observed in vivo, and whilst there is no direct evidence that the change was the result of TGF-β1 expression/activation and the subsequent expression of type I collagen and tenomodulin, it could be hypothesized that these are related. Long-term SB431542 treatment in rats in vivo for 3 weeks may also affect the muscle and other endocrine factors and cause undesired side effects on rat Achilles tendon stiffness [30]. Therefore, in this study, we did not test the effect of SB431542 on inhibition of VV-increased stiffness in rat. Further in vivo studies using tenocyte-specific TGF-β1 knockout mice treated with VV may be useful for confirmed our results that TGF-β1 mediates VV-increased tendon stiffness.

In conclusion, this study determined that low-magnitude, low-frequency 10 Hz VV treatment provides a promising anabolic effect by increasing the expression of TGF-β1, followed by an increased expression of tenomodulin expression and synthesis of type I collagen, improving Achilles tendon stiffness. While vibration training has demonstrated positive results in tendons during exercise and rehabilitation, such as improved mechanical and biochemical tendon properties [10, 51], this study provides insight into the mechanisms by which low-magnitude, low-frequency VV treatment to improve tendon properties, and this treatment method may minimize the risk of ligament/tendon reinjure during rehabilitation.
